# Intellectual Profile of Adolescents with Headache: A Case–Control Study Using the WISC-IV

**DOI:** 10.3389/fneur.2018.00128

**Published:** 2018-03-06

**Authors:** Matteo Chiappedi, Martina Mensi, Eliana Antonaci, Elena Zavani, Livio Tronconi, Cristiano Termine, Umberto Balottin

**Affiliations:** ^1^IRCCS Mondino Foundation, Pavia, Italy; ^2^Department of Brain and Behavioural Sciences, University of Pavia, Pavia, Italy; ^3^Department of Public Health, Experimental and Forensic Medicine, University of Pavia, Pavia, Italy; ^4^Department of Experimental Medicine, University of Insubria, Varese, Italy; ^5^Ospedale di Circolo e Fondazione Macchi, Varese, Italy

**Keywords:** adolescence, headache, intelligence, assessment, Wechsler, migraine, tension-type, WISC-IV

## Abstract

There are few literature evidences about the intellectual profile of adolescents with headache and no study has used the fourth edition of the Wechsler Intelligence Scale for Children (WISC-IV) in patients with a diagnosis of headache according to the ICHD-III-beta. We recruited 30 patients (age 11–14 years; male:female = 1:2) seen for headache in a tertiary center in Northern Italy and 30 healthy controls matched for age and sex, recruited in a public school from the same geographic area. The diagnosis of headache was done according to the ICHD-III criteria (beta version): the case group was composed of 16 patients with migraine and 14 with tension-type headache. Cognitive functioning was assessed using the WISC-IV. Recruited patients with idiopathic headache diagnosis had on average a cognitive function within the normal range. We found no statistically significant differences in the total Intellective Quotient comparing patients with headache and controls; the Working Memory Index was, however, lower in patients with headache (*p* = 0.012), and in particular, we found a lower Digit Span (*p* < 0.001). We also found a borderline statistical difference (*p* = 0.051) between case and controls Verbal Comprehension Index (CVI), which was due to a lower score in the Similarities subtest (*p* < 0.001). Our results suggest that, although within normal limits, cognitive functioning of adolescents with headache differs from that of healthy peers regarding memory and verbal skills. The Working Memory Index is related to the subject’s ability to store new information and keep them in short-term memory, to maintain focused attention and to manipulate them to find solutions. The difference in Similarities is also important because it provides a measure of the level of verbal reasoning and concept formation; it is also a measure of verbal abstract thinking skills relevant for language development, lexical knowledge, auditory comprehension, memory, and ability to discriminate between essential and non-essential characteristics. Our data, in keep with previous findings, suggest the need for further researches to better understand the pathogenesis of these difficulties and obtain ideas for an adequate rehabilitative treatment.

## Introduction

Headache is a common disorder in children and adolescence; it has a negative effect on life quality, is a relevant cause of missed school days, and determines a significant health-related cost (1). The exact prevalence of headache in children and adolescents is debated, but it has been estimated that up to 75% of school-aged children experience occasional headache attacks and about 10% have recurrent episodes (2). Moreover, although the diagnosis is based on pain characteristics and possibly associated symptoms, a number of clinical evidences show that headache causes a complex alteration of the global function of pediatric patients (3).

The correlations between headache, migraine in particular, and cognitive functions have been studied in children and adolescents since 1980. Typically, a reduction in information speed processing has been reported in migraineurs, with a consequent impairment in visual–spatial memory, verbal performances, and attentive skills (4). As to tension-type headache, it is not clear if the difficulties reported in terms of academic failure are due to headache *per se* or to the loss of school days secondary to pain experienced (5). This nonetheless, it is evident that the existing literature concerning cognitive aspects in children and adolescents is far from being conclusive. A number of potentially significant factors have not been evaluated in detail, including psychopathological aspects and the effect of medications taken both for relieving pain and as preventive.

Moreover, published studies have used previous versions of the Wechsler Intelligence Scale while the most recent version published in Italy, i.e., IV edition, is based on a fully different model of intelligence and of cognitive functioning.

As stated by Kamphaus et al. (6), in fact, an intelligence test needs to be designed *a priori*, based on a strong theoretic model and have an at least reasonable evidence of validity in measuring a construct. The Cattell–Horn–Carrol Theory of Cognitive Abilities (7, 8) provided the framework needed for the development of the IV edition of the Wechsler Intelligence Scale for Children (9, 10). The model divides cognitive abilities in wide and narrow ones, all contributing to intelligence as a whole (7, 8).

We decided therefore to conduct a study following these hypotheses:

(1) the existence of cognitive differences between subjects with primary headache and healthy adolescents;
(2) the possibility to define a pattern of cognitive peculiarities according to the Cattell–Horn–Carrol Theory of Cognitive Abilities in patients with primary headache tested with the Wechsler Intelligence Scale for Children—IV edition;
(3) the possibility to associate a specific pattern of cognitive peculiarities to a specific form of primary headache.

## Materials and Methods

### Participants and Procedure

To test the above written hypotheses, we recruited 30 patients with primary headache at the Child and Adolescent Neuropsychiatry Unit, National Neurological Institute IRCCS C. Mondino—University of Pavia (Italy). For all participants in the study, inclusion criteria were being 11–14 years old, not being in a preventive pharmacological treatment, having a form of primary headache, confirming will to participate (informed consent was not only signed by parents or legal guardians but also by the patients). Exclusion criteria were being less than 11 or more than 14 years old, being in a preventive pharmacological treatment, having a secondary form of headache, not willing to participate.

The healthy control group was recruited in a school located in the same city. Subjects were included if they had had no more than one unprovoked headache attack in the last 6 months, as confirmed by an *ad hoc* questionnaire filled by their parents. Subjects with neurological and/or psychiatric disorders, with a personal history of school failure, or with parents refusing to give written informed consent were excluded. 30 subjects were randomly chosen from the eligible ones.

Patients and their parents adhered to the voluntary basis after detailed explanation of the project. Parents and adolescents were informed and expressed their consent to the participation in the study.

### The Wechsler Intelligence Scale for Children—IV Edition

The scale measures intellectual ability of children and adolescents from 6 to 16 years. It was developed to provide an overall measure of general cognitive ability and also measures of intellectual functioning in Verbal Comprehension, Perceptual Reasoning, Working Memory, and Processing Speed. These subscales, respectively, provide scores for the Verbal Comprehension Index (CVI), the Perceptual Reasoning Index, the Working Memory Index, and the Processing Speed Index. Together, the four indexes provide the overall level of intelligence or Full Scale IQ.

Although the full version of the WISC-IV has 15 subtests, only 10 are considered core, and used more often when testing intelligence. The core subtests for Verbal Comprehension are Vocabulary, Similarities, and Comprehension; the core subtests for Perceptual Reasoning are Block Design, Picture Concepts, and Matrix Reasoning; the core subtests for Working Memory are Digit Span and Letter-Number Sequencing; and the core subtests for Perceptual Reasoning are Coding and Symbol Search. We did not exploit any supplementary subtest in any of the adolescents included in our study.

Since it has been reported that headache attacks could have a time-limited effect on cognitive parameters, we decided to administer the WISC-IV after no less than 24 h from the last attack (as reported by the patient by mean of a headache diary) (11).

### Analysis

Descriptive statistics were used to describe the socio-demographic and clinical variables of the participants. After evaluating the distribution of data with the Kolmogorov–Smirnov’s test, the differences between patients and controls were tested using Student’s *t*-test. The same was done to assess differences between patients with migraine and patients with tension-type headache and those with episodic to those with chronic forms [according to ICHD-III-beta criteria (12)] and to compare patients with different duration of the disorder. All differences were considered significant with a level of probability of *p* < 0.05.

## Results

As to the 30 patients with headache, 20 were females (66%) and 10 males (33%). 16 (53%) had migraine [9 (30%) without and 7 (23%) with aura] and 14 (47%) tension-type headache. Duration of headache was more often between 1 and 2 years (21 subjects, 70%), with a minority of patients having headache from less than 1 year (3 subjects, 10%) or more than 2 years (6 subjects, 20%). None of the female patients was taking hormonal contraception; none of the patients declared smoking as a habit [only two male patients declared to have tried tobacco smoking on occasional basis (not more than one time per month)].

The 30 controls were matched in terms of age (mean age cases: 12.4 years, mean age controls: 12.6 years) and sex distribution (exact match).

Table 1 summarizes significant differences between subjects with headache and healthy controls. Recruited patients with idiopathic headache diagnosis had on average a cognitive function within the normal range. We found no statistically significant differences in the total Intellective Quotient comparing patients with headache and controls; the Working Memory Index was, however, lower in patients with headache (*p* = 0.012) and in particular, we found a lower Digit Span (*p* < 0.001). We also found a borderline statistical difference (*p* = 0.051) between case and controls CVI, which was due to a lower score in the Similarities subtest (*p* < 0.001).

**Table 1:** Comparison of Wechsler Intelligence Scale for Children—IV edition scores in subjects with headache and controls.

Score	Controls mean (SD)	Patients mean (SD)	*t* Test (*p*)
Full Scale Intelligence Quotient	117.77 (9.51)	112.80 (13.4)	1.655
Verbal Comprehension Index	120.13 (10.45)	112.87 (17)	1.994
Perceptual Reasoning Index	113.03 (8.94)	113.23 (13.71)	−0.067
Working Memory Index	112.63 (12.9)	104.90 (14.1)	2.620*
Processing Speed Index	104.43 (12.98)	104.80 (13.20)	−0.108
Block Design	11.60 (1.47)	11.23 (2.2)	0.756
Similarities	14.87 (2.31)	12.37 (2.85)	3.722**
Digit Span	12.53 (1.43)	10.27 (2.58)	4.201**
Picture Concepts	12.13 (1.97)	12 (2.39)	0.235
Coding	9.87 (2.19)	10.07 (2.54)	−0.326
Vocabulary	12.1 (2.15)	11.93 (3.03)	0.245
Letter-Number Sequencing	11.87 (1.54)	11.40 (2.52)	58
Matrix Reasoning	12.1 (1.84)	12.5 (2.4)	54,368
Comprehension	12.97 (1.75)	12 (3.56)	58
Symbol Search	11.63 (2.61)	12.17 (3.05)	−0.726

*p < 0.05.

**p < 0.001.

No statistically significant difference was found comparing patients with migraine to patients with tension-type headache, nor comparing those with episodic headache to those with chronic forms. Moreover, we found no statistically significant difference comparing the three groups defined in terms of duration of headache (less than 1 year, between 1 and 2 years, more than 2 years).

## Discussion

To the best of our knowledge, this is the first study assessing cognitive peculiarities in children and adolescents with primary headache using the Wechsler Intelligence Scale for Children—IV edition (and therefore appealing to the Cattell–Horn–Carrol Theory of Cognitive Abilities).

Previously, D’Andrea et al. (13) found a reduction of short- and long-term memory skills in migraineurs compared to age-matched healthy subjects; the study, however, did not find any significant difference in terms of global cognitive functioning. A more recent Brazilian study used the Wechsler Intelligence Scale for Children—III edition to confirm the normality of the Intelligence Quotient in both migraineurs and healthy control, which showed significantly lower scores in some subtests (namely Vocabulary, Information, and Arithmetic) in children and adolescents with headache (14). In order to try to explain these findings, the hypothesis of a decrease of some cognitive skills limited to the migraine attack was studied (15). The authors found some significant differences, but the low number of subjects included and the variability of the changes prevented definite conclusions.

As to tension-type headache, which is the other most common form of primary headache in children and adolescent, a longitudinal study showed that it is a major cause of loss of school days. The authors hypothesized that the decrease in cognitive performance observed in children with headache could be a consequence of the loss of academic opportunities as well as of the reduction in attentive skills and to the negative effect of pain and associated symptoms (5).

An Italian study compared children and adolescent with migraine to those with tension-type headache and to healthy controls by mean of the Wechsler Intelligence Scale for Children—Revised; the authors found a non-significant difference in terms of Intelligence Quotient with healthy subjects overcoming patients with headache (in particular, in subtests examining verbal skills). A significant correlation was observed between the number of attacks and the general Intelligence Quotient, the Verbal Intelligence Quotient, and the Performance Intelligence Quotient (15). This study was replicated by Esposito et al. using the Wechsler Intelligence Scale for Children—III edition; the authors found that the Verbal Intelligence Quotient of patients with tension-type headache was lower and the Performance Intelligence Quotient higher compared to both migraineurs and healthy controls. Moreover, patients with migraine showed a lower Perceptual Organization Index compared to those with tension-type headache (16).

The first point to evidence in our data is that children and adolescents with primary headache have a cognitive functioning which is globally in the normal range. As to the main indexes, the only statistically significant difference in the comparison with a healthy age- and sex-matched group was for the Working Memory Index. This was largely due to a highly significant difference in the subtest Digit Span and is a measure of short-term memory and in particular of working memory, which can be defined as the ability to add new information to existing memories, to store it in the short-term storage system and to effectively and efficiently manipulate it to solve cognitive problems. This is exactly the Gsm ability described in the Cattell–Horn–Carrol Theory of Cognitive Abilities (17). These findings are in keep with already published data (13, 18).

A second relevant difference was found for the subtest Similarities (leading also to a nearly significant difference in terms of CVI). This subtest provides a measure of the level of verbal reasoning and concept formation; it is also a measure of verbal abstract thinking skills relevant for language development, lexical knowledge, auditory comprehension, memory, and ability to discriminate between essential and non-essential characteristics. It is worth remembering that Waldie et al. (18) hypothesized the existence of a not yet identified prenatal factor causing a reduction of verbal abilities, although our study has no potential to add data in this respect.

In keep with the substantial normality of their cognitive evaluation, none of our patients showed significant academic difficulties. We cannot exclude from our findings a possible future deterioration of school achievement in these subjects, also given the possible role of the number of headache attacks in determining a non-regular school frequency and thereof a school failure (5).

Moreover, we found no statistically significant difference between patients and controls in terms of visuo-motor skills; this is far different from what reported by Precenzano and co-workers (19), who, however, showed that this deficit could be remediated through a neuropsychologial rehabilitative intervention.

We found no significant differences between patients with migraine and those with tension-type headache, in keep with data by Parisi et al. (15). The lack of differences between patients with chronic headache compared to those with sporadic headache seems counter-intuitive especially considering the higher burden of other associated difficulties often reported in children and adolescents with chronic headache (20, 21). It is, however, important to evidence that the number of patients in each group was insufficient to allow definite conclusions.

None of our patients was taking preventive drugs at the moment of the test nor had taken painkillers in the 24 h before the test, given that we asked them to be attack-free for at least 24 h before being tested. This is especially important considering the possible cognitive side-effects of drugs, as shown for instance in the recent Children and Adolescent Migrain Prevention study were 17% of subjects taking topiramate experienced memory impairment and 16% a not better specified “cognitive disorder” (22).

Some limitations of this study need to be acknowledged. First, we could not calculate the adequate sample size due to a lack of studies on this topic using the Wechsler Intelligence Scale—IV edition; we decided to have a number of subjects similar to that of studies already published which employed previous versions of the Wechsler Intelligence Scale. Second, we did not assed in detail the emotive functioning of our patients, although psychological peculiarities are frequently seen in adolescents with headache (23, 24) and a certain degree of test-related anxiety has been reported for this group (13). Third, we have no longitudinal data, which would add to the study of cognitive aspects in these patients. Fourth, we did not assess medication overuse, which is, however, a relevant problem and can lead to additional cognitive effects (25, 26). Fifth, the sample size was low, in particular regarding the subgroups so that we cannot exclude an underpowering effect.

Despite these limitations, we believe that our data, in keep with previous findings, suggest the need for further researches to better understand the pathogenesis of the clinically relevant difficulties and to obtain ideas for an adequate treatment, be it pharmacological or based on psychological techniques.

## Ethics Statement

Patients and parents have given their written consent to the participation in the study in accordance with the national and institutional code of good ethical practice and with the Declaration of Helsinki. This study was conducted within the context of a wider research project, approved by the Ethical Committee of National Neurological Institute IRCCS C. Mondino on April 19, 2011.

## Author Contributions

MC and UB wrote the manuscript. MC, MM, and UB contributed to study design, data discussion, and critical evaluation of the manuscript. EZ and EA contributed to data collection and Rorschach scoring. MC performed statistical analyses. CT, LT, and MC contributed to data interpretation. All the authors approved the final manuscript and agreed to be accountable for all the aspects of the study.

## Conflict of Interest Statement

The authors declare that the research was conducted in the absence of any commercial or financial relationships that could be construed as a potential conflict of interest.
